# Ultrahigh-dimensional variable selection method for whole-genome gene-gene interaction analysis

**DOI:** 10.1186/1471-2105-13-72

**Published:** 2012-05-03

**Authors:** Masao Ueki, Gen Tamiya

**Affiliations:** 1 Advanced Molecular Epidemiology Research Institute, Faculty of Medicine, Yamagata University, 2-2-2 Iida-Nishi, Yamagata, Yamagata, Japan

## Abstract

**Background:**

Genome-wide gene-gene interaction analysis using single nucleotide polymorphisms (SNPs) is an attractive way for identification of genetic components that confers susceptibility of human complex diseases. Individual hypothesis testing for SNP-SNP pairs as in common genome-wide association study (GWAS) however involves difficulty in setting overall p-value due to complicated correlation structure, namely, the multiple testing problem that causes unacceptable false negative results. A large number of SNP-SNP pairs than sample size, so-called the large p small n problem, precludes simultaneous analysis using multiple regression. The method that overcomes above issues is thus needed.

**Results:**

We adopt an up-to-date method for ultrahigh-dimensional variable selection termed the sure independence screening (SIS) for appropriate handling of numerous number of SNP-SNP interactions by including them as predictor variables in logistic regression. We propose ranking strategy using promising dummy coding methods and following variable selection procedure in the SIS method suitably modified for gene-gene interaction analysis. We also implemented the procedures in a software program, EPISIS, using the cost-effective GPGPU (General-purpose computing on graphics processing units) technology. EPISIS can complete exhaustive search for SNP-SNP interactions in standard GWAS dataset within several hours. The proposed method works successfully in simulation experiments and in application to real WTCCC (Wellcome Trust Case–control Consortium) data.

**Conclusions:**

Based on the machine-learning principle, the proposed method gives powerful and flexible genome-wide search for various patterns of gene-gene interaction.

## Background

Single SNP association study is a popular method to detect genes that are susceptible to human diseases. Although candidate gene approach that uses prior knowledge about its function is an efficient procedure, it could overlook genes whose function is unknown or ambiguous. GWAS using whole genome SNPs is thus an attractive solution to this issue. Many procedures proposed for GWAS so far are based on marginal association between each SNP and phenotype. However, it has been pointed out that most susceptible-SNPs identified often show low or moderate effect size, and hence may explain only a few percentage of the genetic variance [1]. The fact that, for several complex diseases, the recurrence risk ratio decreases quicker than 1/2 as the relatedness decreases implies the involvement of non-additive interactions in their etiology [2,3]. In addition to substantial contributions of rarer variants, gene-gene and gene-environment interaction would be one of strong candidates that can explain the missing heritability as well [1]. Without investigating such interactions, we therefore may miss genuine disease-susceptible loci [4,5]. An effective and accurate method to search gene-gene interactions will utilize immediately original SNP-GWAS data to decipher such missing genetic components of complex human diseases. In this paper, we tackle a development of powerful method for the genome-wide gene-gene interaction analysis using SNPs.

Marchinni et al. [4] suggest that the use of arbitrary single locus-disease association model under which some interaction effect is present might prevent from finding susceptible interaction effect. Therefore exhaustive search for interactions are needed rather than primary screening by marginal effect. Although individual hypothesis tests for all SNP-SNP pairs as frequently used in SNP-GWAS might be the simplest approach, it is however faced with multiple testing issues caused by 300,000-1,000,000 SNPs. Specifically, there is a difficulty in setting genome-wide significance level using, e.g., Bonferroni correction, FPRP [6], or Bayes factor [7,8], leading to prohibitively conservative result because they fail to successfully incorporate the correlation structure between each hypothesis. For instance, the total number of hypothesises for second-order gene-gene interaction is about 10^11^ –10^12^ in standard GWAS data. Then Bonferroni-corrected significance level must be considerably small by multiplying the nominal significance level such as 0.01 by the correction factor less than 10^−11^. No efficient and universal multiple testing method to deal with such the huge set of hypotheses having complicated correlation structure is proposed so far. Although multiple logistic regression may be the method that can incorporate the correlation structure between hypothesises by putting them as predictor variables, the so-called large *p* small *n* situation (i.e. the number of predictors is larger than the sample size), precludes simultaneous inclusion of all SNP-SNP interactions in the logistic regression model, namely, no unique solution is determined.

There are several software programs for gene-gene interaction analysis including multifactor dimensionality reduction (MDR) [9], fast-epistasis option in PLINK [10], Tuning Relief [11], Random Jungle [12], BEAM [13], and BOOST [14]. Cordell [5] carried out an extensive comparative study for these methods. She reported that MDR and BEAM have computational difficulty in analyzing current scale in GWAS and that random Jungle is applicable to small scale dataset but not for whole-genome dataset. PLINK-fast-epistasis and BOOST are designed for exhaustive search. Since both methods are based on hypothesis testing principles, as stated above, exact determination of significance level for whole-genome data is difficult due to the numerous multiple hypotheses in addition to the complicated correlation structure between them. Hypothesis testing methods [4,15,16] could be too conservative and lead to false negative result, because some complicated correlation structure would commonly appear when considering interaction pairs. The BOOST has been shown to outperform the PLINK--fast-epistasis in most of the interaction models considered in [14]. The BOOST primarily performs screening of possible hypotheses using Kirkwood superposition approximation (KSA), then applies standard multiple testing on the basis of the initial number of SNP-SNP pairs to the likelihood ratio statistic under the saturated logistic regression model having the degrees of freedom (df) of 4. However, the test statistic may fail to follow the chi-squared distribution of df 4 under the presence of sparse cells, which causes too small type 1 error rate [14]. Because the sparse cell issue is unavoidable in genotypic interaction data, some solutions in development of summary statistic for gene-gene interaction are strongly necessitated. Our method is free from the sparse cell issue owing to the proposed dummy coding strategy, which generates 2 by 2 contingency table.

In this paper we develop a new method based on contemporary ultrahigh-dimensional variable selection approach, designated the sure independence screening (SIS) [17-19] rather than hypothesis testing. The principle of SIS is based on the same as that of machine learning methods [5,9] exploring the optimal model that can explain sufficiently the data in the most parsimonious way; its methodological feature to avoid over- or under-fitting enables us flexibly to capture various patterns of gene-gene interaction. SIS is such a simple combination of marginal regression and following penalized multiple regression [17-19] that can become just an effective framework for the analysis of SNP-SNP interaction, which forms a typical ultrahigh-dimensional data In multiple regression model, regression coefficients can be used for evaluation of the importance of predictors, i.e. coefficients closer to zero indicate less contribution to the regression formula. Statistical evaluation that infers regression coefficients to be zero is referred to as the variable selection [20], which may lead to multiple regression model consisting of fewer predictors with improved estimation accuracy. Recent progress has justified theoretically and empirically the usefulness of variable selection in the large *p* small *n* situation [21-23]. Wu et al. [24], Hoggart et al. [25], and Ayers and Cordell [26] have been proposed applications of the modern variable selection to the selection of effective SNPs in single SNP association study. Actually, SIS has already been used in single SNP association analysis in GWAS data [27]. Recently, SIS is theoretically proven to accept exponential grows of parameters as the increase of sample sizes in generalized linear models [19]. This capability of exponentially large number of predictors relative to sample size encourages us the advanced application of the SIS to the genome-wide gene-gene interaction analysis. In its application, there is a difficulty in defining predictors for SIS ranking step that can represent the interaction effect for each pair because the combination of two SNPs forms a 3 by 3 contingency table for which multiple patterns of interaction models are conceivable. The ranking step is very important to effectively capture various patterns of interactions. In this paper we elaborate the ranking strategy by proposing a promising three dummy coding methods and following variable selection procedure which is suitable for SNP-SNP interaction analysis. We also implemented the proposed method in a very fast program, EPISIS. This is the first software program to employ the ultrahigh-dimensional variable selection method that can provide a statistically valid and high-speed exhaustive SNP-SNP interaction analysis even for standard GWAS. The application studies to simulated datasets show that the proposed method works successfully and accurately. EPISIS was applied to find some novel genetic components in the WTCCC (Wellcome Trust Case–control Consortium) data.

## Results and discussion

### Simulation experiments

We carried out simulation experiments to examine power and type 1 error rate for the proposed EPISIS using the same dataset as those used in Wan et al. [14] for BOOST.

#### Power

**Figure 1:**
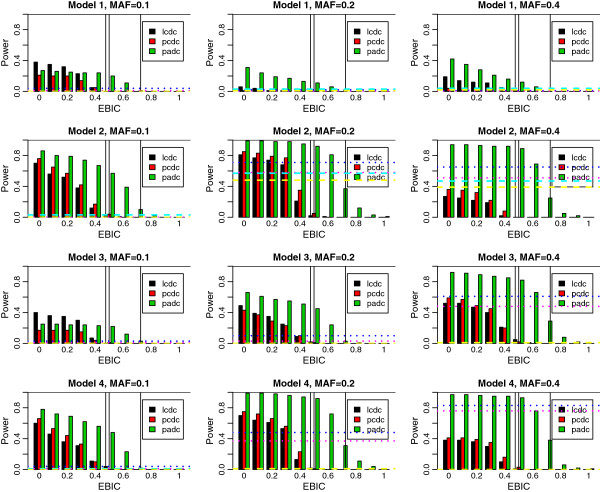
**Power simulation for scenarios 1–12 in 400/400 case–control data with LCDC, PCDC, and PADC. **The dotted horizontal lines show power resulted from BOOST with significance levels 5% (pink) and 30% (blue) after Bonferroni correction. The dashed horizontal lines show power resulted from PLINK --fast-epistasis with significance levels 5% (yellow) and 30% (cyan) after Bonferroni correction. Three vertical lines emphasize LCDC and PCDC at γ = 0.5 and PADC at γ = 0.7, respectively.

**Figure 2:**
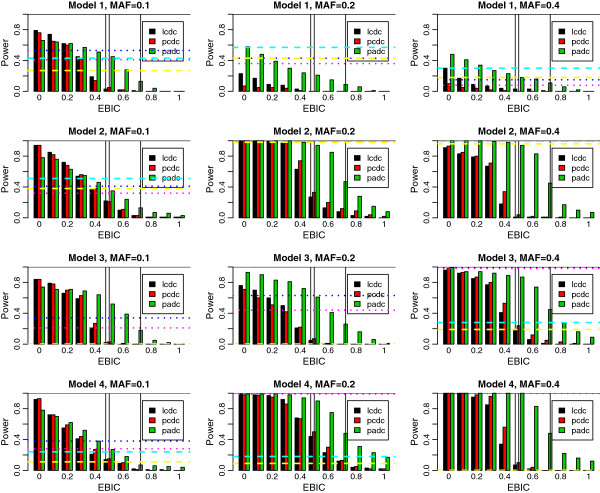
**Power simulation for scenarios 1–12 in 800/800 case–control data with LCDC, PCDC, and PADC. **The dotted horizontal lines show power resulted from BOOST with significance levels 5% (pink) and 30% (blue). Three vertical lines emphasize LCDC and PCDC at γ = 0.5 and PADC at γ = 0.7, respectively.

To investigate the power, we use the simulated data available from the BOOST website (http://bioinformatics.ust.hk/BOOST.html), which allows us a power comparison between EPISIS, BOOST, and PLINK--fast-epistasis. Details of the datasets are summarized in Additional file 1: Text S1. Here we note that the power comparison between BOOST and PLINK--fast-epistasis has already been given by [14]. The datasets were simulated from 12 different interaction models (scenarios 1–12) based on the four epistatic interaction patterns (models 1–4) and three different MAFs, 0.1, 0.2, and 0.4. First three of the 12 scenarios are generated from model 1 with three MAFs (scenarios 1–3), next three are from model 2 (scenarios 4–6), and so on. Each dataset contains 100 replicates with 1,000 SNPs in 400/400 or 800/800 case–control individuals, among which the pair of first and last SNPs is set to the disease-susceptible combination and remaining 998 SNPs are non-risk factors. Power is calculated as the proportion that the interaction pair is detected in 100 replicates. Figures 1 and 2 (400/400 and 800/800) show the power results for three coding methods, likelihood cell-wise dummy coding (LCDC), *p*-value cell-wise dummy coding (PCDC), and *p*-value adaptive dummy coding (PADC), where the abscissa corresponds to the extended Bayesian information criterion (EBIC) tuning parameters γ between [0,1] at an interval of 0.1, i.e. 11 equally spaced points. (The detailed explanation for EBIC is given in Method section.) For comparison we applied the BOOST and PLINK –fast-epistasis for the same datasets. The power results from these methods with significance levels of 5% and 30% are also shown in Figures 1 and 2, calculated by the output of BOOST’s *p*-value applying Bonferroni correction on the basis of the number of interactions based on the number of SNPs, namely, we use the Bonferroni correction with *s*(*s*-1)/2 hypotheses for *s* SNPs. The BIC (EBIC at γ = 0) results in most powerful, then the power declines asγ goes to 1. Later, we discuss about the power carefully in terms of the balance with the type 1 error rate. The 12 panels in Figures 1 and 2 are arranged in the same order of the Figure 2 of [14] for ease of comparison. Because no versatile calibration method for γ has been proposed so far, we use the type 1 error rates resulted from simulated datasets as a guide for reasonable choice of γ.

#### Type 1 error rate

We calculate the type 1 error rate as 1- (proportion that no factors are detected in replicates). We use the two scenarios given in [14]. Scenario 1: we generated 1,000 replicates where no LD exists among SNPs using PLINK --simulate option, each having 1,000 SNPs whose MAFs are uniformly distributed within the interval [0.05,0.5] and including 500 case and 500 control individuals. Scenario 2: we generated 100 replicates where LD exists among SNPs using genomeSIMLA [28] on the basis of the marker information on the Affymetrix 500Â K chip from human chromosome 1, where each dataset contains 38,836 SNPs in 500 case and 500 control samples.

The resulting type 1 error rates for EPISIS are summarized in Table 1 and those for PLINK –fast-epistasis and BOOST given in Additional file 2: Table S1 are close to the results in [14]. In all simulations, BIC (EBIC at γ = 0) shows large type 1 error, and EBIC at some γ > 0 leads to no type 1 error for all methods. In summary, there exists a trade-off between power and type 1 error rate. From the type 1 error rates in scenario 1 (Table 1) corresponding to the null version of the power simulations, we can select 0.4 for LCDC and PCDC, and 0.6 for PADC, at which the type 1 error rate is roughly comparable to the nominal error rates 10-30% adopted in Figure 2 of [14]. Turning to the power plots at these EBIC values, the PADC shows highest power compared with the LCDC and PCDC, and outperforms BOOST except for cases 6, 9, and 11 in 400/400 samples, and in cases 3, 7, and 10 for 800/800 samples, which implies that the EPISIS works effectively on the models that the BOOST shows low power. On the other hand for the models on which the EPISIS results in inferior performance to BOOST, the observation that the difference from BOOST in power is modest suggests that our proposed method can work effectively for several kinds of interaction patterns. The optimal EBIC values in scenario 2 were larger by 0.1 than that in scenario 1 for three coding methods, LCDC, PCDC, and PADC, which may come from the existence of LD or the fact that the number of SNPs before screening in scenario 2 is larger than that in scenario 1.

**Table 1:** Summary of type 1 error simulations of EPISIS

	**γ**	^**0**^	^**0.1**^	^**0.2**^	^**0.3**^	^**0.4**^	^**0.5**^	^**0.6**^	^**0.7**^	^**0.8**^	^**0.9**^	^**1**^
	^LCDC^	^1^	^0.997^	^0.965^	^0.895^	^0.098^	^0^	^0^	^0^	^0^	^0^	^0^
^No LD^	^PCDC^	^1^	^1^	^0.991^	^0.143^	^0.143^	^0^	^0^	^0^	^0^	^0^	^0^
	^PADC^	^1^	^1^	^1^	^0.998^	^0.985^	^0.204^	^0.002^	^0^	^0^	^0^	^0^
	^LCDC^	^1^	^1^	^1^	^0.96^	^0.86^	^0.08^	^0^	^0^	^0^	^0^	^0^
^LD^	^PCDC^	^1^	^1^	^1^	^0.96^	^0.81^	^0.07^	^0^	^0^	^0^	^0^	^0^
	^PADC^	^1^	^1^	^1^	^1^	^0.99^	^0.97^	^0.83^	^0.04^	^0^	^0^	^0^

Type 1 error rates in scenarios 1 (no LD) and 2 (LD) with varying γ_._

Although it is impossible to complete simulation experiments in a genome-wide scale within realistic computational time, we adopt γ to be 0.4 or 0.5 for LCDC and PCDC, and 0.6 or 0.7 for PADC, in the following WTCCC data analysis, so that both power and type 1 error rate are of practical use as suggested by our simulation studies.

### WTCCC data analysis

We applied our program EPISIS to real GWAS datasets provided from WTCCC (the Wellcome Trust Case Control Consortium), which included about 500,000 common SNP genotypes for each 2,000 cases of seven human diseases, bipolar disorder (BD), coronary artery disease (CAD), Crohn’s disease (CD), hypertension (HT), rheumatoid arthritis (RA), type 1 and type 2 diabetes (T1D and T2D), with 3,000 controls [29]. We first made high quality SNP datasets through a standard quality-control filter, MAF in control > 0.05, Hardy-Weinberg Exact (HWE) test *p*-value < 5.7e-7, study-wise missing data proportion > 0.05, 1df Trend Test or 2df General Test *p*-values < 5.7e-7 between two control populations. After eliminating SNPs without flag of “good-clustering” on signal summary information, for these diseases, we finally had datasets consisting of 357,320 SNP genotypes that were processed in our program.

For an exhaustive search of all possible two-pair interactions in addition to their main effects, on average, we needed about seven hours to complete one search on a standard LINUX machine having four GPU units (NVIDIA Tesla C2050). We carried out three search strategies described in Method section for each dataset using three different combinations of two ranking measures and two coding methods, i.e. 1) CDC with likelihood (LCDC), 2) CDC with *p*-value (PCDC), and 3) ADC with *p*-value (PADC). The details are described in Method section.

We gathered the annotated information about each SNP in our results from annotations released by Affymetrix (R31). As summarized in Table 2, through these exhaustive searches, we finally obtained 1) seven interactions for BD, one for CAD, one for CD, 37 for HT, 23 for RA, two for T1D and zero for T2D using LCDC (γ = 0.4), 2) two for BD, zero for CAD, one for CD, two for HT, one for RA, one for T1D and zero for T2D using PCDC (γ = 0.4), 3) zero for BD, zero for CAD, five for CD, zero for HT, one for RA, one for T1D and one for T2D using PADC (γ = 0.6). All of our results are listed in Additional file 3: Table S2. Among surviving variables through these searches, one main effect (rs6679677 in 1p13.2) for RA and two (rs9272723 and rs9272346 in HLA class II region) for T1D were detected by LCDC as well as PCDC, which were found in the original report [29]. In contrast, PADC reported no main effect that was stronger than interactions. We found a large number of interactions between SNPs located within a single gene locus (i.e. dominance effect) particularly in BD, HT and RA when using LCDC.

**Table 2:** Summary of interactions from EPISIS for seven WTCCC diseases

	**LCDC**					**PCDC**					**PADC**				
	E^a^	D^b^	M^c^	Total^d^	N^e^	E	D	M	Total	N	E	D	M	Total	N
BD	2	5	0	7	6	1	1	0	2	2	0	0	0	0	0
CAD	1	0	0	1	1	0	0	0	0	0	0	0	0	0	0
CD	1	0	0	1	1	1	0	0	1	1	5	0	0	5	3
HT	8	29	0	37	17	2	0	0	2	2	0	0	0	0	0
RA	14	8	1	23	9	0	0	1	1	-	1	0	0	1	1
T1D	0	0	2	2	-	0	0	1	1	-	2	0	0	1	1
T2D	0	0	0	0	0	0	0	0	0	0	0	0	0	0	0
Sub Total	26	42	3			4	1	2			8	0	0		

^a^Epistasis; ^b^Dominance effect between SNPs in narrow (<100Â kb) region or same locus; ^c^Main effect; ^d^Total number of second-order interactions; ^e^Network group of interactions.

**Figure 3:**
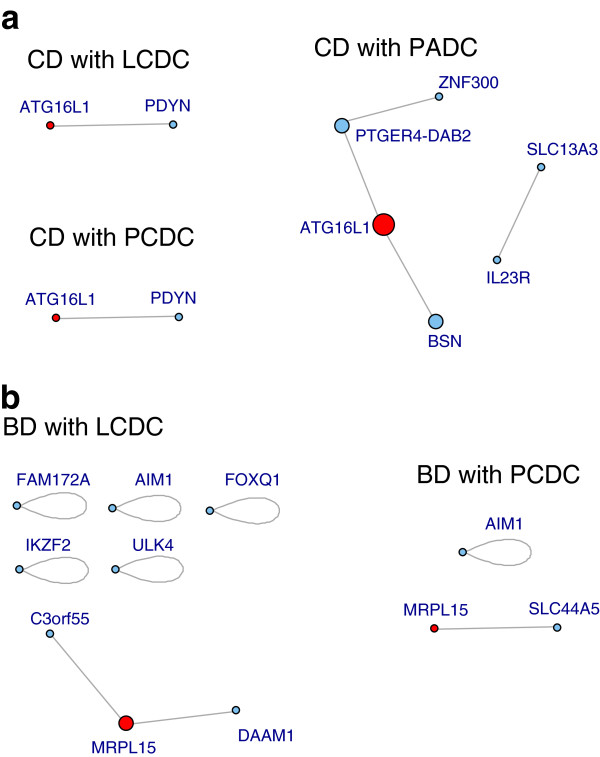
**SNP network graphs assembled for SNP-SNP interactions by three methods, LCDC, PCDC and PADC in CD. **Circle and line represents “node” and “edge”, respectively. Red circles represent SNPs in ATG16L1 or MRPL15.

We detected the greatest number of interactions in HT by LCDC, BD and HT by PCDC, and CD by PADC. However, they included many redundant interactions that was shared a same SNP as a partner. For example, although PADC reported five interactions in CD (Additional file 4: Table S3), those interactions shared same SNPs that can be assembled into two “network groups” implying potential higher-order interactions (Figure 3).

Three methods in EPISIS, LCDC, PCDC, and PADC, yielded apparently quite different results in number of detected interactions, but by expressing as gene networks it turns out that they report similar gene-gene interactions in addition to some otherwise extras. For example, in CD (Figure 3a), some interesting interactions including a gene showing main effect were reported; that between ATG16L1 (autophagy-related protein 16-like 1) on 2q37.1 and PDYN on 20p13 (OR_CDC_(95%CI) = 1.76 (1.542–2.008)) in addition to ATG16L1 - PTGER4 (prostaglandin E receptor 4) - ZNF300 (zinc finger protein 300) (ATG16L1 - PTGER4: OR_CDC_(95%CI) = 2(1.7–2.4); PTGER4 – ZNF300: OR_ADC_(95%CI) = 1.7(1.5–1.9)) and so on. LCDC appears to have a tendency to report a greater number of interactions than other two methods that utilize *p*-value ranking, and the difference was emphasized in dominance effects. Throughout our data analysis, the *p*-value ranking tends to be more conservative than the likelihood ranking. (We argue the phenomenon in Method section.) On the other hand, CDCs appear to have a tendency to report dominance effects in addition to epistatic effects. This phenomenon may be accounted for by the fact that the dominance effects are observed between linked loci, and so increases the sparsity of the 3 by 3 genotype contingency table because of the concentration to the diagonal cells (i.e. aa-bb, aA-bB or AA-BB). A typical one of the examples is the second SNP-SNP pair found in the result for BD with the LCDC (rs2438083 - rs977673) given in Additional file 3: Table S2 online, where an apparent difference between cases and controls captured by the LCDC is seen in the aA-BB cell while other cells have very small number of observations due to the increased sparstiy. As implied by the power simulations, CDC can work well for capturing disease-development models that suffice with a single cell, and therefore is applicable to the dominance effect mentioned above.

In CD, most of the interactions shared a SNP in ATG16L1, which is an autophagy related gene showing the strongest main effect (*p* < 10^−13^) originally reported in WTCCC paper. It is not always necessary that genes involving in interactions show strong main effect. In another type of disease, BD, no strong main effect was reported, but EPISIS detected a substantial number of interactions (Table 2). Range of odds ratio (95%CI) detected was between 0.01(0.000–0.93) and 7.25(4.007–13.12). LCDC and PCDC reported a gene-gene interaction around MRPL15 (Figure 3b).

## Conclusion

We proposed an effective method for gene-gene interaction analysis using SNPs and developed a fast software program EPISIS enabling genome-wide gene-gene interaction analysis by utilizing the cost-effective GPGPU technology. This is the first method that successfully implements ultrahigh-dimensional variable selection approach for an exhaustive search for gene-gene interactions using realistic scale of SNP-GWAS data. The method implemented employs a framework of SIS [17-19], which enables us to handle huge set of SNP-SNP interactions based on the modern large *p* small *n* regression methodology, rather than prohibitively conservative methods through conventional hypothesis testing.

Simulation studies describe that our EPISIS show successful performance. Among three ranking strategies proposed, PADC showed greater performance than LCDC and PCDC in terms of power for most scenarios. The exceptions exist in models 2 with MAF = 0.1 (n = 1600) and model 4 with MAF = 0.1 (n = 1600), which may be well approximated by the disease-development model that suffices with a single cell of the 3 by 3 genotype interaction table due to the low MAFs. Indeed, the CDC frequently captured the major homozogotes pair (i.e. AA and BB cell) in both two simulations. Intuitively, the CDC is workable for the interaction pattern influenced by a single or a few cells, while the ADC is appealing to more complicated interaction patterns since the ADC strengthens summary statistic of interaction by gathering cell-wise dummy predictors of CDC in an adaptive manner. Following the power simulation studies, we recommend using PADC as a main method and CDC as a useful complement because the CDC may be of help in certain situations. Thus, we recommend using PADC as a main method and CDCs as complements. The power analysis through simulated datasets reveals that PADC captures various interaction patterns, including the models for which BOOST resulted in low power, in practically high performance. On the other hand, there exist some models where PADC shows modestly lower power than BOOST but still maintains practicable performance. Our studies therefore show that the PADC can have practically high power for several diseases models, which is a result from the use of analogous technique used in MDR by classifying SNP-SNP genotype pairs in two groups (high-risk and low-risk groups). Notably, since our proposed strategies generate 2 by 2 contingency table based on dummy coding, the sparse cell issue present in the BOOST is resolved.

One remaining issue in EPISIS is that the control of type 1 error rates is somewhat rough, which is common in variable selection approaches, although it brings flexibility of the method. The proposed choice of EBIC parameter depends on the simulated datasets (scenarios 1 and 2), which are less than genome-wide scale. In addition, we observed modest increase of type 1 error rates under presence of linkage disequilibrium (LD) compared with the simulation under no LD. This observation is explained by a well-known fact that stringent correlation worsens performance of multiple regression. Nevertheless, Additional file 5: Figure S1 shows that LCDC and PCDC at γ = 0.4 or PADC at γ = 0.6 in the application to seven real WTCCC data can reduce the number of detected predictors as seen in the simulation studies, which implies that these EBIC values for each screening method become a realistic solution. Here we note that the information about the number of detected predictors as varying γ may give us a further candidate gene sets. Indeed, the power plots imply that the smaller γ successfully increases the chance to include genuine interaction in the SIS ranking. We therefore encourage calibrating γ to be able to have the interaction pairs ranked high on the list, which could include susceptible loci to be examined in confirmatory study. Such a flexible usage is one of the advantages in using machine learning-based methods.

By applying EPISIS to WTCCC datasets, we obtained a large number of interactions potentially conferring susceptibility to them. The distributions of the interactions detected EPISIS were disease-specific but not software- or method-specific, implying that these results were likely derived due to the genuine distributions of these interaction but not pseudo-negative and/or positive due to the algorithm in EPISIS. Although a confirmatory study using independent sample set is needed to eliminate bias and confounding specific to original data, some of these interactions assembled in an interpretable network graph appear to be plausible from their functional points of view. For example, a statistical interaction among ATG16L1/PTGER4/ZNF300 in CD could imply the involvement of a synergistic combination among autophagy and other possible mechanisms such as immune-inflammation in the etiology of inflammatory bowel disease, which have been repeatedly suggested by genetic studies about their main effects [29-32] as well as mechanistic studies [33,34]. In contrast, one network of interactions found in BD centered MRPL15 connected to many other genes of which some are possibly involved in mitochondrial function. This finding seems to support the previous hypothesis that mitochondrial (dys)functions underlies the pathophysiology of mental illnesses such as bipolar disorder and schizophrenia [35,36]. EPISIS also reported a large number of interactions between neighboring SNPs, which are expected to have remarkable dominance effects through potential haplotype-blocks, which might confer disease susceptibility as haplotype blocks consisted of SNPs in a single locus.

**Figure 4:**
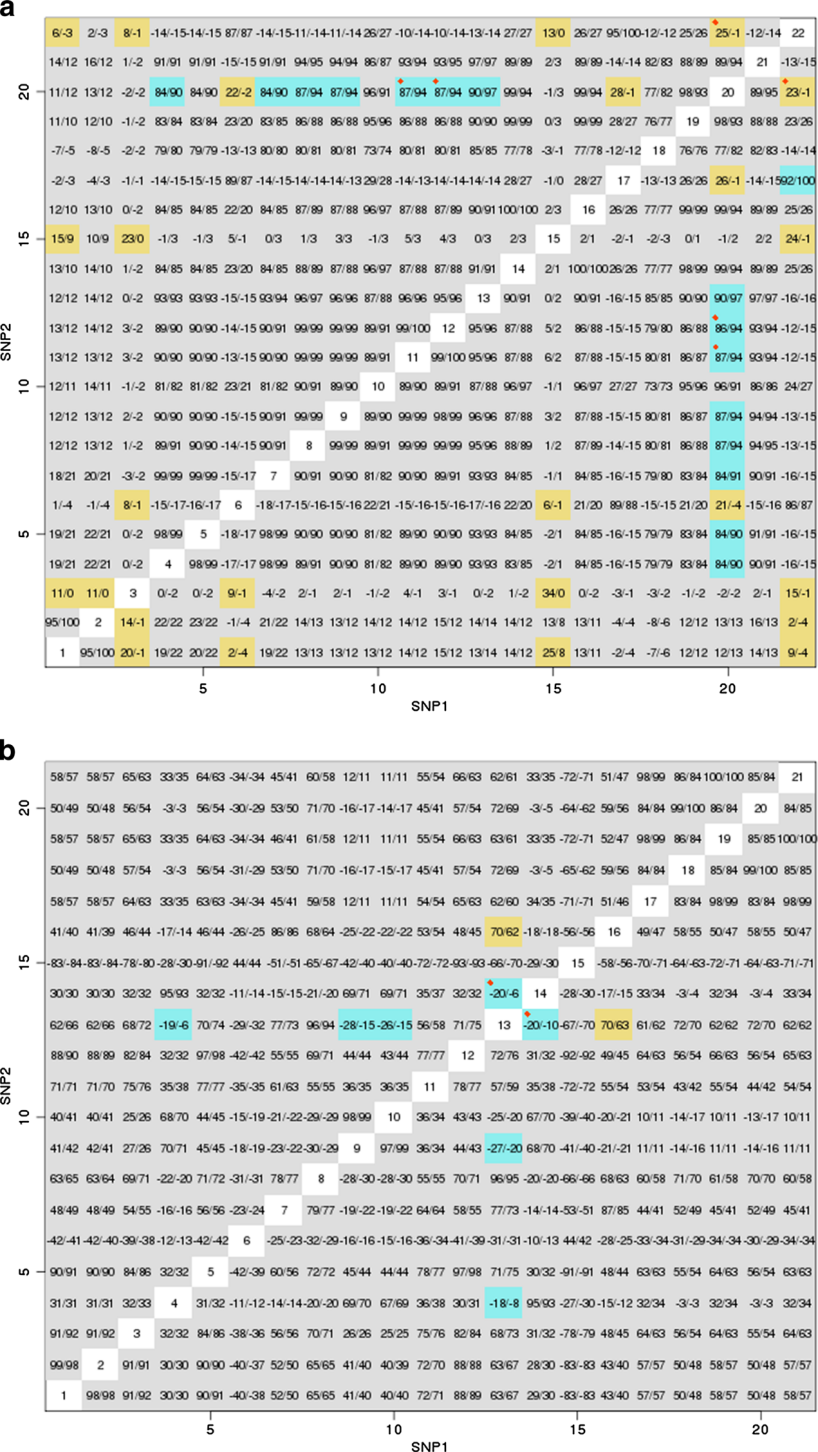
**Correlation matrix for PPM1A and ULK4. **(**a**) PPM1A and (**b**) ULK4, Upper- and lower triangle parts are haplotype- and genotype-based correlation coefficients for cases/controls (%), respectively. Pairs are highlighted if the difference from cases to controls is less than −0.06 (skyblue) and is larger than 0.06 (gold), SNP-SNP pairs detected by EPISIS are emphasized by red diamond symbol. SNPs are coded as follows: (**a**) 1. rs17093348, 2. rs6573295, 3. rs4901972, 4. rs11849674, 5. rs10148587, 6. rs188620, 7. rs10137732, 8. rs6573298, 9. rs7145505, 10. rs8019531, 11. rs11628587, 12. rs11628628, 13. rs8011227, 14. rs7158657, 15. rs17097243, 16. rs10142834, 17. rs17097262, 18. rs1887104, 19. rs1887103, 20. rs7154773, 21. rs8012816, 22. rs10130695; (**b**) 1. rs9311275, 2. rs9832048, 3. rs9852385, 4. rs13069584, 5. rs7627972, 6. rs2632594, 7. rs9812025, 8. rs6599155, 9. rs12054014, 10. rs12054016, 11. rs7649806, 12. rs6794510, 13. rs6809441, 14. rs33916626, 15. rs11129908, 16. rs7618902, 17. rs4973952, 18. rs9878069, 19. rs1495696, 20. rs1495698, 21. rs9834824.

For illustrative purpose, we examined the LD pattern of the dominance effect detected by EPISIS using two genes, PPM1A and ULK4, as an example, which were found in our analysis for HT. As stated above, the EPISIS attempts to detect the difference between case- and control-distributions. To see this in detail, we provide in Figure 4 the correlation coefficients based on haplotype and genotype frequencies, which can represent a distributional characteristic. The genotype-based correlation coefficient is proposed by Wellek and Ziegler [37]. To compare the difference between cases and controls, Wellek and Ziegler’s statistic is more appropriate than the haplotype-based correlation coefficient, since the estimation of haplotype frequencies requires the condition in which Hardy-Weinberg equilibrium holds, which is not expected in case population. Since the purpose here is to compare case- and control-distributions, consideration of the sign is needed. Figure 4 describes the correlation coefficient matrix for cases and controls for PPM1A (a) and ULK4 (b). Haplotype-based and genotype-based correlations are given in the upper- and lower-triangle parts, respectively. SNP-SNP pairs that EPISIS detected are emphasized by red diamond symbol. It can be seen that all pairs are included in pairs highlighted in the figure.

Here we compare our results in WTCCC data analysis with those using BOOST and PLINK –fast-epistasis respectively given by Wan et al. [14] and Cordell [5]. First, since exhaustive search using PLINK –fast-epistasis is virtually infeasible due to computational burden, Cordell [5] conducts semi-exhaustive search for CD based on SNPs that passed the single locus *p*-value threshold of 0.2. She concluded that the SNP-SNP pair that showed highly significant fast-epistasis *p*-value is false positive. On the other hand, EPISIS detected a few interactions for CD in LCDC, PCDC, and PADC as shown in Additional file 4: Table S3. The analysis using EPISIS contains no interactions located in top 16 using PLINK given in Additional file 2: Table S1 of [5]. This difference comes from the semi-exhaustive search used in [5] as well as the fact that the summary statistic in fast-epistasis is based on allelic correlation whereas that in EPISIS is based on genotypic data. Second, Wan et al. [14] report many gene-gene interactions detected by the BOOST for T1D in the MHC (major histocompatibility complex) region, although they mention that the interactions found for other six diseases are nontrivial except for one SNP pair in CD (the details are not shown in their paper). They state that the interactions obtained in T1D analysis after excluding significant loci under single-locus scan include interesting interaction patterns between the MHC class I and class II. Although our EPISIS finds some interactions at two SNPs in the MHC class II, rs9272723 and rs9272346, these two SNPs show strong main effect and are interacted with SNPs on other chromosome, i.e. not in the MHC class I. This discrepancy from the BOOST’s result could be partly explained by the fact that we analyzed entire SNPs without imposing single-locus significance threshold. Although it could be possible to exclude SNPs showing strong main effect, we prefer including them since they contribute forming the affected and unaffected populations and could be ancillary to interaction analysis through regression model. The EPISIS’s result on T1D tells that the SNPs in the MHC class II region can play a major role in partitioning affected and unaffected individuals even when considering second-order interactions. It is also noteworthy that the interaction detected by EPISIS include various SNP-SNP pairs having cells with fewer observations (Additional file 3: Table S2), implying that the EPISIS overcomes the sparse cell issue. Since BOOST, PLINK –fast-epistasis, and EPISIS have advantage and disadvantage depending on disease patterns that underlie as seen in power simulation, we recommend using them in a mutually complementary manner.

Finally, we summarize our future works in what follows. First, since we use the multiple regression model, inclusion of covariates is possible to adjust the influence of several confounding factors, such as sex, age, or population stratification. Second, the generalized linear model used in SIS allows extension to more general phenotypes other than disease status. Third, although the current version does not allow the presence of missing genotype in the future version, we would overcome this issue. Fourth, an iterative version of SIS (ISIS) has been proposed [17,18], which could improve the detection ability. We have already implemented the ISIS in EPISIS, which is useful in finding additional interactions confounded by interactions that have already detected. It poses further computational cost due to increased dimensionality of parameters, where the cost depends on the number of detected factors in SIS and subsequent penalized regression. An issue arises when detected dummy predictors after SIS are highly-correlated, i.e. multi-collinearlity, causing failure in convergence of estimating regression coefficients in SIS ranking step, which incurs considerable increase of computational time while some of results lose their reliability. Unfortunately, we provide no reasonable solution to this issue and it is still under consideration. ISIS could work if no multi-collinearlity exists, although we have no knowledge about relationship between type 1 error rates and EBIC tuning parameters, with extra predictors provided from the previous SIS step, in penalized regression step. Consequently, at the moment, we have to pay adequate attention to the multi-collinearity within the dummy predictors as well as to the selection of EBIC tuning parameter in using ISIS procedure, and we will address such issues in our future work. Although the ISIS where the above issues are resolved is ultimately desirable, the current EPISIS will work reasonably as in simple situations used in the simulation studies.

Our program EPISIS described in this manuscript will be freely available from the authors’ webpage soon.

## Methods

### High-dimensional multiple regression and variable selection

We first review the high/ultrahigh-dimensional variable selection, and then describe our method used in EPISIS. It is well known that the speed of convergence of regression coefficients is 1/*n*^1/2^ as function of sample size *n*. However, the argument holds only when the number of predictors *p* is fixed or small compared with *n*. Huber [38] and Portony [39] obtained a refined result regarding estimation accuracy of regression coefficients as (*p*/*n*)^1/2^. This implies that larger *p* is never preferable as it worsens the estimation accuracy. When we handle large number of predictors, i.e. high- and ultrahigh-dimensional data, conventional multiple regression analysis can fail. A possible solution to this issue is derived from assuming that some of predictors are redundant and do not contribute to the response variable. The corresponding regression coefficients are then set to zero, i.e. the variable selection, which explores an optimal model among competitors using an appropriate selection criterion including AIC [40], BIC [41], cross-validation [42], GCV [43], and Mallows’ C_p_[44]. Shao [45] investigated several theoretical properties regarding them.

Exhaustive search of the possible candidates is virtually infeasible when *p* is large, because the number of candidate models is 2^*p*^. Lasso [21,22] and elastic net [23] were proposed in order to address the issue and to complete the variable selection even in such a high-dimensional data. In genome-wide association study, the application of lasso logistic regression [24] and hyper-lasso [25] has been proposed. Ayers and Cordell [26] conduct extensive simulation studies to compare various variable selection methods for several thousands of candidate SNPs. Variable selection has been applied not to gene-gene interaction analysis but to single SNPs association study so far. On the other hand, although coordinate decent-type method [25,26,46,47] is computationally more efficient than Lars-type algorithm [22,48], it will be still hard to complete the computation in realistic time when considering genome-wide gene-gene interaction. The sequential update also prevents from getting faster using parallel computing. Furthermore, the requirement of selecting tuning parameters makes it difficult in use. To overcome the issue, we employ the SIS method [17-19] that is the most computationally efficient variable selection method that is feasible for genome-wide gene-gene interaction analysis.

#### Sure independence screening

The SIS [17-19] is the simplest method relatively to the other variable selection procedures. It first looks marginal association using the univariate regression independently, hence is suited to parallel computing. The advantage of SIS is that favorable theoretical properties have been established [19]. Theoretically, SIS can detect effective predictors even in ultrahigh-dimensional situation [19]. To be specific, it allows the number of predictors *p* such that $\text{log}p=o(n^{1-2\mathrm{Îº}})$, where *Îº* is some constant between 0 and 0.5. This allows handling exponentially large number of predictors, typically in genome-wide interaction search. Main assumptions in SIS are (i) Number of nonzero parameters are few, and (ii). Marginal covariance between effective predictors and response variables is larger than some but not stringent threshold value. (Note that the “marginal” indicates the single predictors rather than the single SNPs.) Fan and Song’s paper [19] should be consulted for detailed and rigorous technical conditions of original SIS. Since their theory assumes generalized linear models, the SIS method is applicable to case–control studies using logistic regression model. The SIS consists of two steps, feature ranking and penalized regression in a similar fashion to the forward–backward stepwise procedure in multiple regression.

The aim of variable selection is to find indices of zero regression coefficients in the *p* dimensional linear function:

$$g\{E(Y)\}=\beta_{0}+\beta_{1}X_{1}+\beta_{2}X_{2}+\cdots +\beta_{p}X_{p}\text{,}$$

where, *Y* is the response variables, *g* is a link function that maps mean of response variable *E*(*Y*) to a linear space, *X*_*j*_ represents the *j*th predictor variable, β_*j*_ is the corresponding regression coefficients, and β_0_ is the intercept. In logistic regression with canonical link, *g* corresponds to the logistic transformation, and the slope β_*j*_ corresponds to logarithm of odds ratio. The total number of possible ways of assigning zeros to the regression coefficients is 2^*p*^. The description of the two steps is as follows.

*Feature ranking step*:

(i). Create ranking for *p* predictor variables based on marginal evaluation through univariate regression for response regressed by*X*_*j*_,

(ii). Extract top *d* = 256 predictor variables from ranking obtained from (i).

*Penalized regression step*: *d* = 256 predictor variables extracted in feature ranking step are validated and well-selected through the smooth-thresholding logistic regression method. Due to computational limitation, *d* is restricted to be less than 256, while Fan et al. [18] recommend d=⌊0.25n/logn⌋. The restriction is however not essential, because the range of *d* falls within 146–271 when *n* = 5000–10000, respectively, makes the restriction to 256 reasonable in terms of current scale of GWA studies.

### EPISIS: Feature ranking step

The important task to apply the SIS to gene-gene interaction analysis is to design predictor variables *X*_*j*_ in logistic regression model. A main contribution of our work is to propose a method that generates effective dummy variables designed for capturing SNP-SNP interaction effect. Dummy coding corresponds to the 2 by 2 table, which equivalently assigns the high- and low-risk group for each cell in 3 by 3 genotype 1nteraction table, which is used in MDR [9]. The strategy thus attempts to maximize the difference between case- and control- distributions. The 2 by 2 table representation makes the interpretation easier through the familiar odds ratio. Moreover, it enables to avoid instability caused by the presence of sparse cells as in MDR. We propose two strategies for designing dummy predictor variables, cell-wise dummy coding (CDC) and adaptive dummy coding (ADC).

#### Cell-wise dummy coding (CDC)

CDC is a cell-wise evaluation for 3 by 3 SNP-SNP genotypic interaction table. The procedure is analogous to the Sham and Curtis’s [49] T_3_ method. Their method, however, applies to the single highly-polymorphic locus association study in order to overcome the invalidity of chi-squared test for a large and sparse contingency table. Let we have two loci 1 and 2 that consist of alleles A/a and B/b, respectively. Then, we can have 9 dummy variables for each pair of SNPs as follows.

$$\begin{array}{l} X_{1}=I(\text{locusÂ }1=aa\text{Â andlocusÂ }2=bb),\\ X_{2}=I(\text{locusÂ }1=aa\text{Â andÂ locusÂ }2=bB),\\ X_{3}=I(\text{locusÂ }1=aa\text{Â andÂ locusÂ }2=BB),\\ X_{4}=I(\text{locusÂ }1=aA\text{Â andÂ locusÂ }2=bb),\\ X_{5}=I(\text{locusÂ }1=aA\text{Â andÂ locusÂ }2=bB),\\ X_{6}=I(\text{locusÂ }1=aA\text{Â andÂ locusÂ }2=BB),\\ X_{7}=I(\text{locusÂ }1=AA\text{Â andÂ locusÂ }2=bb),\\ X_{8}=I(\text{locusÂ }1=AA\text{Â andÂ locusÂ }2=bB),\\ X_{9}=I(\text{locusÂ }1=AA\text{Â andÂ locusÂ }2=BB)\text{,} \end{array}$$

where *I*() denotes the indicator function. After a repetition of the above procedure, 9 *L*(*L*-1)/2 dummy variables are generated for *L* SNPs. For diseases showing no significant interactions, consideration of main effect in addition to interaction can lead to proper conclusion. We therefore consider simultaneously single SNPs main effect in addition to computation for SNP-SNP interactions. For a locus 1 with alleles A/a, we introduce three dummy variables corresponding to three genotypes

$$\begin{array}{l} X_{1}=I(\text{locusÂ }1=aa),\\ X_{2}=I(\text{locusÂ }1=aA),\\ X_{3}=I(\text{locusÂ }1=AA)\text{.} \end{array}$$

Adding these three predictor variables to the above 9 *L*(*L*-1)/2 predictors pertaining to the interactions, we finally have 9 *L*(*L*-1)/2 + 3 *L* predictors in total and create feature ranking for them. Consequently, it allows equal evaluation between interaction and main effects, and enables to sort them according to their strength of the effect through the ranking.

*Remarks*: Since CDC creates nine dummy variables, the occurrence of multi-collinearlity might be suspected. This is true only if all nine dummy predictors in a pair of SNPs appear in the top 256 in SIS ranking. However, it is unrealistic when the number of loci is large because if one of nine cells shows larger odds ratio relative to the others, the one of the other cells must have smaller odds ratio, consequently resulting that it does not appear in the ranking and in the further variable selection part.

#### Adaptive dummy coding (ADC)

An alternative way is to define the dummy variable that represents a pair of SNPs.

ADC employs a principle similar to that used in the MDR [9] so that categorizes nine cells into the high-risk and low-risk groups. There are two remarkable differences between ADC and MDR.

1) Exhaustive comparison of possible classifications.

2) The ADC compares 2^9^-2 classifications of dummy coding for each pair of two SNPs that separate high-risk and low-risk groups.

3) (There are 2^9^ ways of separating nine cells to two classes; -2 indicates elimination of two cases with only high-risk and low-risk group.)

4) Evaluation of classification accuracy using original dataset.

5) We evaluate the result of classification using the original dataset.

6) We use the balanced accuracy (BA), which is shown to perform well in the MDR method [50], as a criterion to evaluate the goodness of classification.

7) The formula is given by BA = (Sensitivity + Specificity)/2.

8) BA is also known as the area under the curve (AUC) of the receiver operating characteristic (ROC) that can be also used as a measure of the potential discriminatory power.

The advantage of taking (i) is as follows. Original MDR method generates two groups, high- and low-risk groups, based on the ratio of case–control individuals exceeding unity for each cell of nine pairs (empty cells are exception), which can sometimes cause false positive and negative errors in some situations [51]. The odds ratio [51] is a criterion alternative to the ratio of case–control individuals, however, it could generate missclassification provided that the sample sizes of either cases or controls are small for a specific cell. Furthermore, threshold value common in both measures is difficult to determine. The above arguments deduces that the entire search of possible 2^9^-2 classifications is preferable to avoid missing the best classification in the marginal SNP-SNP interaction table

On the other hand, regarding (ii), the original MDR uses the cross-validated data to evaluate the classification accuracy instead of original dataset. Although the use of cross-validated dataset is a careful approach, it incurs computational burden in genome-wide gene-gene interaction analysis. The main purpose of this feature ranking is to make a mere list of candidates of genuine classification of the two SNPs combination toward the next penalized regression step but not to infer them. In our procedure, the subsequent penalized regression plays a role of adequate model validation instead of the cross-validation.

#### Association criterion

We propose two criteria for feature ranking step: *p*-value of odds ratio and likelihood for the 2 by 2 contingency table corresponding to the dummy coding. Originally, Fan and their colleagues [17,18] use the absolute slope in logistic regression obtained from univariate regression. In logistic regression model, this slope corresponds to the odds ratio. In the 2 by 2 contingency table, it is known that the odds ratio is more sensitive to the presence of small number of observations as is usual. Thus, instead of odds ratio itself, we use its *p*-value. If zero cells appear in the table, we add 0.5 to all cells of the observed counts for calculations of *p*-value and likelihood.

From the test statistic point of view, *p*-value of odds ratio and likelihood ranking are asymptotically equivalent, because the former and latter correspond to the Wald- and likelihood ratio test statistics for testing the slope of zero in univariate logistic regression model. However, they can differ in finite sample situation. Indeed, the real data analysis of the GWAS data from WTCCC described in the following section shows that interactions detected by the likelihood ranking have larger odds ratio than those by the *p*-value ranking, while the corresponding *p*-values of odds ratio are of course smaller than those by *p*-value ranking. Since the odds ratio is popularly used in epidemiology, possibly due to the relationship with relative risk, the *p*-value may provide more interpretable result than likelihood criterion.

#### Comparison between CDC and ADC

CDC allows inclusion of two or more dummy variables in each pair of SNPs, while ADC does only one dummy variable. Thus, ADC is effective for some diseases of which the difference of frequencies between cases and controls can be sufficiently represented by some high- and low-risk classification. For otherwise diseases, CDC will work well, in other words, it is preferable under more complicated situation. Inclusion of more than two predictors in a pair in CDC also loses the chance that the interactions in boundary in the ranking of 256 predictors are included, while the ADC does not occupy 256 rankings by the same pair. ADC carries out re-partitioning of nine cells into two cell categories, i.e. susceptible or not, leading to increase of the number of samples in each category, so that ADC method is likely to be more stable than CDC.

#### Gene-gene interaction

As mentioned above, a major aim of our feature ranking is to sort SNP-SNP pairs according to the extent of difference between case- and control-distributions, so it takes into no account of distances between two SNPs, possibly leading to strong LD between them. Indeed, our feature ranking from an exhaustive search may include not only pairs of which two SNPs are less-correlated but also those which are highly-correlated. The former clearly corresponds to epistatic interactions between genes represented by each SNP, while the latter might indicate interactions between SNPs located within a narrow region (frequently within a single gene locus), nearly analogous to the conventional concept of “dominance” effect. In fact, focusing on statistical relationship of two SNPs is merely equivalent to mathematically considering 3 by 3 contingency tables of genotype pairs (for cases and controls). Since then, statistical closeness between two SNPs in the table is simply evaluated through some correlation measures such as the Pearson’s correlation coefficients regardless of their physical distance, gene-gene interaction between unlinked regions and that in the same region are indistinguishable at least in the mathematical model as well as in the context of the conventional quantitative genetics, i.e. non-additive effects [52]. Even if any pair of SNPs in a narrow region showing considerable LD are unavoidably appeared in the ranking, it will turn out that the EPISIS effectively detect potential haplotype-blocks consisted of these SNPs in LD that show strong interaction effect but no large marginal association of each SNP. Although epistasis and dominance may have distinct mechanisms differentiating distributions of frequency of genotype pairs between cases and controls, EPISIS may detect any interactions irrespective of either cause.

### Penalized regression step

For this step, we employ the smooth-thresholding developed by one of us [53,54] that applies to the ridge logistic regression method, termed the ridge smooth-thresholding logistic regression. We explain the procedure in detail in what follows. Suppose that *d* predictor variables are survived after feature ranking step. Let ${\hat{\beta }}_{j}^{\left(0\right)}$ be an initial estimate for the *j*th regression coefficient, and let, for given tuning parameter *λ*, the index set be $A\left(\lambda \right)=\left\{j\in \left\{1,\ldots ,d\right\}:|{\hat{\beta }}_{j}^{\left(0\right)}|\leq \lambda \right\}$ Then, the ridge smooth-thresholding logistic regression minimizes the following criterion with respect to β_*j*_ for all *j*ε*A*(*λ*) the active set,

$$\begin{array}{l} \ell (\beta )+\frac{1}{2}\Sigma_{j\varepsilon A(\lambda )}\left\{\lambda_{2}+\frac{\delta_{j}(\lambda )}{1-\delta_{j}(\lambda )}\right\}\beta_{j}^{2},\\ \delta_{j}(\lambda )=\min(1,\lambda /|{\hat{\beta }}_{j}^{\left(0\right)}{|}^{2})\text{,} \end{array}$$

in which ℓ(β) is the log-likelihood function of logistic regression with regression coefficients β_*j*_, *j*ε{1, …, *d*}. Here *λ*_2_ denote the ridge tuning parameter. Regression coefficients β_*j*_ not belonging to *A*(*λ*) are set to zero, i.e. sparse solution. We utilize the ridge logistic regression estimates for the initial estimates ${\hat{\beta }}_{j}^{\left(0\right)}$, of which the ridge tuning parameter *λ*_2_ is determined in the following manner: If the matrix inversion in Newton–Raphson iteration step fails, we increase the ridge parameter until it first succeeds. The initial *λ*_2_ is set to 1/*n*. Notably, the ridge smooth-thresholding logistic regression is closely related with the adaptive elastic net [55], which has been applied not to the logistic regression but to least-squares regression.

Although the extension to logistic regression is straightforward, the required convex programming in the adaptive elastic net incurs computational instability in obtaining the solution to the logistic regression estimation such as the failure of convergence, while the smooth-thresholding is free from the issue using simpler Newton–Raphson procedure. Convex programming is required by most of variable selection methods including SCAD [56,57], MCP [58], Adaptive Lasso [59], Lasso [21,22], Elastic net [23], Adaptive elastic net [55], and Dantzig selector [60,61]. Consequently, the smooth-thresholding is favorable over other methods in the present purpose. The ridge penalty *λ*_2_ plays a role in stabilizing the variable selection process when multi-collinearlity is present, which is useful because dummy predictor variables extracted from the feature ranking step often include members that are highly-correlated, possibly due to LD. We select the tuning parameters *λ* using the extended BIC [62] which control the number of survived predictors.

Screening step can bring additional variability in selected predictors, which may fail direct application of the BIC to them according to the recent studies [62,63] pointing out that large number of predictors increases the chance of detecting false positives in variable selection when using the BIC. With dimension of the saturated model *p*, the extended BIC for a model having an estimate $\hat{\beta }$ is defined as follows.

$$\text{EBIC}=-2\ell (\hat{\beta })+(\text{log}n+2\gamma \;\text{log}\;p)\dim(\hat{\beta })\text{,}$$

where γ is a given constant taking its value within the interval [0,1]. EBIC at γ = 0 reduces to the BIC, and the increase of γ imposes heavier penalty for dimensionality which decreases type 1 error rate. We take the dimension *p* not the number of screened predictors but the number of predictors before screening step in order to take into account for the variability due to screening. For CDC, *p* is 9 *L*(*L*-1)/2 + 3 *L*, while *p* is for ADC *L*(*L*-1)/2. EBIC may be conservative if choosing γ = 1 because the penalized regression is applied to the screened predictors not to the full predictors. Since, unfortunately, no universal method to choosing γ has been proposed so far, simulation studies offer a guide for the choice. Other selection criteria such as AIC and GCV are not recommended in use for variable selection purpose since they tend to select variables more than truth [64].

## Competing interests

The authors declare that they have no competing interest.

## Authors’ contributions

MU and GT carried out the simulation study and the real data analysis. MU is responsible for the algorithm of proposed method. They drafted the manuscript and approved the final manuscript.

## Supplementary Material

Additional file 1 **Text S1.** Details of simulated datasets in power simulation studies. Click here for file

Additional file 2 **Table S1. **Type 1 error rates for PLINK –fast-epistasis and BOOST in scenarios 1 and 2. Click here for file

Additional file 3 **Table S2. **EPISIS output file. Click here for file

Additional file 4 **Table S3. **Two-order interactions from episis for Crohn’s disease and bipolar disorder. Click here for file

Additional file 5 **Figure S1. **Number of detected interactions varying EBIC tuning parameter in application to seven WTCCC datasets. Click here for file
