# Supplementary material for: Ultrahigh-dimensional variable selection method for whole-genome gene-gene interaction analysis
Source: BMC Bioinformatics. 2012 May 3;13:72. doi: 10.1186/1471-2105-13-72 (PMC3531267; doi:10.1186/1471-2105-13-72)
Supplement: Additional file 5 — Figure S1. Number of detected interactions varying EBIC tuning parameter in application to seven WTCCC datasets. [file 1471-2105-13-72-S5.doc]

Supplementary Text 1

**Simulated datasets in power simulation**

We used the simulated datasets available from <http://bioinformatics.ust.hk/BOOST.html>, "Case 1 -- Disease loci with main effects", which is used in Wan et al. (2010 Am J Hum Genet). The sample sizes are 800 (400 cases/400 controls) and 1600 (800 cases/800 controls), and 1000 SNPs are included in which SNP2 - SNP999 are of non-risk alleles whereas the interaction of SNP1 and SNP1000 is of risk factor according to four epistatic interaction models. 100 replication samples are included for all settings.

**1. Details of four interaction models**

Odds tables are described as follows, where "a" and "A" represent minor and major alleles for SNP1 and "b" and "B" represent those for SNP1000, respectively.

**Model 1**

|  | bb | bB | BB |
| --- | --- | --- | --- |
| aa | $\alpha(1+\theta)^{4}$ | $\alpha(1+\theta)^{2}$ | $\alpha$ |
| aA | $\alpha(1+\theta)^{2}$ | $\alpha(1+\theta)$ | $\alpha$ |
| AA | $\alpha$ | $\alpha$ | $\alpha$ |

The simulated data are generated from the model 1 for three minor allele frequencies (MAF) of 0.1, 0.2, 0.4 with parameters $(\alpha,\theta)$:

MAF = 0.1, $(\alpha,\theta)$ = (0.099888304248452, 3.451551551551551)

MAF = 0.2, $(\alpha,\theta)$ = (0.091234687715769, 1.298708708708709)

MAF = 0.4, $(\alpha,\theta)$ = (0.074684154242277, 0.624384384384384)

**Model 2**

|  | bb | bB | BB |
| --- | --- | --- | --- |
| aa | $\alpha$ | $\alpha$ | $\alpha(1+\theta)$ |
| aA | $\alpha$ | $\alpha$ | $\alpha(1+\theta)$ |
| AA | $\alpha(1+\theta)$ | $\alpha(1+\theta)$ | $\alpha$ |

The simulated data are generated from the model 1 for three minor allele frequencies (MAF) of 0.1, 0.2, 0.4 with parameters $(\alpha,\theta)$:

MAF = 0.1, $(\alpha,\theta)$ = (0.077234348282218, 1.538468468468469)

MAF = 0.2, $(\alpha,\theta)$ = (0.064732041209936, 1.638368368368368)

MAF = 0.4, $(\alpha,\theta)$ = (0.064732041209936, 1.639604568481445)

**Model 3**

|  | bb | bB | BB |
| --- | --- | --- | --- |
| aa | $\alpha$ | $\alpha$ | $\alpha(1+\theta)$ |
| aA | $\alpha$ | $\alpha(1+\theta)$ | $\alpha$ |
| AA | $\alpha(1+\theta)$ | $\alpha$ | $\alpha$ |

The simulated data are generated from the model 1 for three minor allele frequencies (MAF) of 0.1, 0.2, 0.4 with parameters $(\alpha,\theta)$:

MAF = 0.1, $(\alpha,\theta)$ = (0.099389115348458, 3.066936936936937)

MAF = 0.2, $(\alpha,\theta)$ = (0.089247827418149, 1.793213213213213)

MAF = 0.4, $(\alpha,\theta)$ = (0.074293231591582, 1.533473473473474)

**Model 4**

|  | bb | bB | BB |
| --- | --- | --- | --- |
| aa | $\alpha$ | $\alpha(1+\theta)$ | $\alpha$ |
| aA | $\alpha(1+\theta)$ | $\alpha$ | $\alpha(1+\theta)$ |
| AA | $\alpha$ | $\alpha(1+\theta)$ | $\alpha$ |

The simulated data are generated from the model 1 for three minor allele frequencies (MAF) of 0.1, 0.2, 0.4 with parameters $(\alpha,\theta)$:

MAF = 0.1, $(\alpha,\theta)$ = (0.078189652413130, 1.538468468468469)

MAF = 0.2, $(\alpha,\theta)$ = (0.066922511905432, 1.603403403403404)

MAF = 0.4, $(\alpha,\theta)$ = (0.061142491176724, 1.718288288288288)

**2. LD status among non-risk SNPs (SNP2 - SNP999)**

For 998 non-risk SNPs (SNP2 – SNP999), we evaluate the LD status through D’ and r^2 for the simulated datasets across 100 replicates. We calculate the minimum, 1^st^ quantile, median, mean, 3^rd^ quantile, maximum of D’ and r^2 for 998 * (998-1)/2 pairs from 998 non-risk SNPs and the following table displays means over 100 replicates for each simulation scenarios. For all scenarios, the tables show absence of LD among the non-risk SNPs.

| n=800 | MAF | Stat | Min | 1st.Qu | Median | Mean | 3rd.Qu | Max |
| --- | --- | --- | --- | --- | --- | --- | --- | --- |
| Model 1 | 0.1 | D' | 2.49E-17 | 2.16E-02 | 4.78E-02 | 7.32E-02 | 9.20E-02 | 1.00E+00 |
|  |  | r^2 | 3.15E-34 | 1.28E-04 | 5.74E-04 | 1.26E-03 | 1.67E-03 | 3.00E-02 |
|  | 0.2 | D' | 2.49E-17 | 2.16E-02 | 4.77E-02 | 7.31E-02 | 9.19E-02 | 1.00E+00 |
|  |  | r^2 | 3.12E-34 | 1.28E-04 | 5.73E-04 | 1.26E-03 | 1.67E-03 | 3.02E-02 |
|  | 0.4 | D' | 2.35E-17 | 2.16E-02 | 4.77E-02 | 7.29E-02 | 9.18E-02 | 1.00E+00 |
|  |  | r^2 | 3.11E-34 | 1.28E-04 | 5.73E-04 | 1.26E-03 | 1.67E-03 | 3.01E-02 |
| Model 2 | 0.1 | D' | 2.43E-17 | 2.16E-02 | 4.78E-02 | 7.31E-02 | 9.19E-02 | 1.00E+00 |
|  |  | r^2 | 3.05E-34 | 1.28E-04 | 5.74E-04 | 1.26E-03 | 1.67E-03 | 3.03E-02 |
|  | 0.2 | D' | 2.49E-17 | 2.16E-02 | 4.78E-02 | 7.31E-02 | 9.19E-02 | 1.00E+00 |
|  |  | r^2 | 3.17E-34 | 1.28E-04 | 5.73E-04 | 1.26E-03 | 1.67E-03 | 3.02E-02 |
|  | 0.4 | D' | 2.51E-17 | 2.16E-02 | 4.77E-02 | 7.29E-02 | 9.18E-02 | 1.00E+00 |
|  |  | r^2 | 3.18E-34 | 1.28E-04 | 5.74E-04 | 1.26E-03 | 1.67E-03 | 3.07E-02 |
| Model 3 | 0.1 | D' | 2.53E-17 | 2.17E-02 | 4.79E-02 | 7.34E-02 | 9.22E-02 | 1.00E+00 |
|  |  | r^2 | 3.05E-34 | 1.28E-04 | 5.74E-04 | 1.26E-03 | 1.67E-03 | 3.07E-02 |
|  | 0.2 | D' | 2.51E-17 | 2.16E-02 | 4.77E-02 | 7.29E-02 | 9.17E-02 | 1.00E+00 |
|  |  | r^2 | 3.17E-34 | 1.28E-04 | 5.73E-04 | 1.26E-03 | 1.67E-03 | 3.01E-02 |
|  | 0.4 | D' | 2.55E-17 | 2.16E-02 | 4.77E-02 | 7.29E-02 | 9.17E-02 | 1.00E+00 |
|  |  | r^2 | 3.13E-34 | 1.28E-04 | 5.73E-04 | 1.26E-03 | 1.67E-03 | 3.03E-02 |
| Model 4 | 0.1 | D' | 2.51E-17 | 2.16E-02 | 4.78E-02 | 7.31E-02 | 9.19E-02 | 1.00E+00 |
|  |  | r^2 | 3.12E-34 | 1.28E-04 | 5.73E-04 | 1.26E-03 | 1.67E-03 | 3.01E-02 |
|  | 0.2 | D' | 2.40E-17 | 2.16E-02 | 4.77E-02 | 7.30E-02 | 9.18E-02 | 1.00E+00 |
|  |  | r^2 | 3.13E-34 | 1.28E-04 | 5.73E-04 | 1.26E-03 | 1.67E-03 | 3.06E-02 |
|  | 0.4 | D' | 2.41E-17 | 2.16E-02 | 4.77E-02 | 7.30E-02 | 9.18E-02 | 1.00E+00 |
|  |  | r^2 | 3.09E-34 | 1.28E-04 | 5.73E-04 | 1.26E-03 | 1.67E-03 | 3.00E-02 |

| n=1600 | MAF | Stat | Min | 1st.Qu | Median | Mean | 3rd.Qu | Max |
| --- | --- | --- | --- | --- | --- | --- | --- | --- |
| Model 1 | 0.1 | D' | 3.27E-17 | 1.52E-02 | 3.36E-02 | 5.13E-02 | 6.46E-02 | 1.00E+00 |
|  |  | r^2 | 3.48E-34 | 6.38E-05 | 2.86E-04 | 6.28E-04 | 8.30E-04 | 1.52E-02 |
|  | 0.2 | D' | 3.13E-17 | 1.52E-02 | 3.36E-02 | 5.13E-02 | 6.46E-02 | 1.00E+00 |
|  |  | r^2 | 3.63E-34 | 6.37E-05 | 2.85E-04 | 6.27E-04 | 8.30E-04 | 1.51E-02 |
|  | 0.4 | D' | 3.01E-17 | 1.52E-02 | 3.37E-02 | 5.15E-02 | 6.48E-02 | 1.00E+00 |
|  |  | r^2 | 3.48E-34 | 6.38E-05 | 2.86E-04 | 6.28E-04 | 8.30E-04 | 1.47E-02 |
| Model 2 | 0.1 | D' | 3.16E-17 | 1.53E-02 | 3.38E-02 | 5.17E-02 | 6.50E-02 | 1.00E+00 |
|  |  | r^2 | 3.51E-34 | 6.37E-05 | 2.86E-04 | 6.27E-04 | 8.30E-04 | 1.53E-02 |
|  | 0.2 | D' | 3.03E-17 | 1.52E-02 | 3.36E-02 | 5.13E-02 | 6.46E-02 | 1.00E+00 |
|  |  | r^2 | 3.39E-34 | 6.38E-05 | 2.86E-04 | 6.27E-04 | 8.31E-04 | 1.51E-02 |
|  | 0.4 | D' | 3.03E-17 | 1.52E-02 | 3.36E-02 | 5.14E-02 | 6.47E-02 | 1.00E+00 |
|  |  | r^2 | 3.48E-34 | 6.38E-05 | 2.86E-04 | 6.28E-04 | 8.30E-04 | 1.50E-02 |
| Model 3 | 0.1 | D' | 3.14E-17 | 1.52E-02 | 3.37E-02 | 5.15E-02 | 6.48E-02 | 1.00E+00 |
|  |  | r^2 | 3.65E-34 | 6.38E-05 | 2.86E-04 | 6.28E-04 | 8.31E-04 | 1.52E-02 |
|  | 0.2 | D' | 3.11E-17 | 1.52E-02 | 3.36E-02 | 5.13E-02 | 6.46E-02 | 1.00E+00 |
|  |  | r^2 | 3.57E-34 | 6.37E-05 | 2.85E-04 | 6.27E-04 | 8.30E-04 | 1.52E-02 |
|  | 0.4 | D' | 2.90E-17 | 1.52E-02 | 3.36E-02 | 5.12E-02 | 6.45E-02 | 1.00E+00 |
|  |  | r^2 | 3.36E-34 | 6.38E-05 | 2.86E-04 | 6.27E-04 | 8.30E-04 | 1.52E-02 |
| Model 4 | 0.1 | D' | 3.00E-17 | 1.52E-02 | 3.37E-02 | 5.14E-02 | 6.47E-02 | 1.00E+00 |
|  |  | r^2 | 3.73E-34 | 6.38E-05 | 2.86E-04 | 6.27E-04 | 8.30E-04 | 1.51E-02 |
|  | 0.2 | D' | 3.03E-17 | 1.52E-02 | 3.36E-02 | 5.12E-02 | 6.45E-02 | 1.00E+00 |
|  |  | r^2 | 3.63E-34 | 6.36E-05 | 2.85E-04 | 6.27E-04 | 8.30E-04 | 1.48E-02 |
|  | 0.4 | D' | 3.09E-17 | 1.52E-02 | 3.37E-02 | 5.14E-02 | 6.47E-02 | 1.00E+00 |
|  |  | r^2 | 3.64E-34 | 6.37E-05 | 2.85E-04 | 6.27E-04 | 8.30E-04 | 1.50E-02 |
